# Association of Diagnosis of Leukodystrophy With Race and Ethnicity Among Pediatric and Adolescent Patients

**DOI:** 10.1001/jamanetworkopen.2018.5031

**Published:** 2018-11-21

**Authors:** Joshua L. Bonkowsky, Jacob Wilkes, Tyler Bardsley, Veronica M. Urbik, Greg Stoddard

**Affiliations:** 1 Brain and Spine Center, Primary Children’s Hospital, Salt Lake City, Utah; 2 Department of Pediatrics, University of Utah School of Medicine, Salt Lake City; 3 Intermountain Healthcare, Salt Lake City, Utah; 4 School of Medicine, University of Utah School of Medicine, Salt Lake City; 5 Department of Internal Medicine, University of Utah School of Medicine, Salt Lake City

## Abstract

**Importance:**

Inherited leukodystrophies are a group of neurological diseases affecting myelin that cause significant morbidities and death. Timely and correct diagnosis is important for initiating treatment, designing disease screening, and offering care and guidance to patients and families.

**Objective:**

To determine whether there are disparities in leukodystrophy diagnosis in different racial backgrounds.

**Design, Setting, and Participants:**

This case-control study involved a retrospective review of patients aged 18 years or younger who were diagnosed with 1 of 4 leukodystrophies (metachromatic leukodystrophy, X-linked adrenoleukodystrophy, Krabbe disease, and Hurler disease) in the US Children’s Hospital Association’s Pediatric Health Information System database from October 1, 2015, through September 30, 2017.

**Main Outcomes and Measures:**

Leukodystrophy diagnosis and racial background of the patients were analyzed. Adjusted prevalence estimates of leukodystrophies were obtained by controlling for sex, insurance type, urban or rural status, 2010 median household income for patient zip code, number of inpatient days, and age at first visit. Pathogenic leukodystrophy gene allele frequencies in different racial backgrounds for *ABCD1*, *ARSA*, *GALC*, and *IDUA* were determined using the gnomAD database.

**Results:**

Of the 557 patients identified with a leukodystrophy (221 [40%] female; 321 [58%] white non-Hispanic, 54 [10%] black non-Hispanic, and 51 [9%] white Hispanic; median [range] age, 7 [0-18] years), nonwhite race, including black non-Hispanic, black Hispanic, and white Hispanic, was associated with not having a leukodystrophy diagnosis. The adjusted prevalence for a leukodystrophy diagnosis in white non-Hispanic patients was 13.8 (95% CI, 10.6-17.9) per 100 000 patients, compared with 5.8 (95% CI, 3.8-8.9), 2.4 (95% CI, 1.1-5.2), and 7.4 (95% CI, 5.2-10.4) per 100 000 in black non-Hispanic, black Hispanic, and white Hispanic patients, respectively. This reduced rate of diagnosis was out of proportion to the frequency of the different races in the Pediatric Health Information System database. Similar or higher frequencies of missense or loss-of-function alleles were measured in populations of Latino and African descent for the pathogenic leukodystrophy gene alleles. For example, for *ABCD1*, allele frequencies in those of Latino or African descent were 2.1 × 10^−5^ and 2.2 × 10^−5^, as compared with 1.4 × 10^−5^ for those of European non-Finnish descent.

**Conclusions and Relevance:**

Patients of racial/ethnic minorities, including those from black, black Hispanic, and white Hispanic backgrounds, were significantly less likely to be diagnosed with a leukodystrophy. Leukodystrophy disease–associated allele frequencies were the same or higher in populations of Latino or African descent, arguing against a genetic founder effect being responsible for the lower diagnosis rates. This underdiagnosis has implications for newborn screening programs and treatment access and may reflect a more widespread problem in pediatric neurological and orphan diseases.

## Key Points

**Question:**

Are there disparities in leukodystrophy diagnosis in patients of different racial and ethnic backgrounds?

**Findings:**

This case-control study of 557 patients with a diagnosis of leukodystrophy found that US patients of racial/ethnic minorities, including those from black, black Hispanic, and white Hispanic backgrounds, were significantly less likely to be diagnosed with a leukodystrophy. Furthermore, leukodystrophy disease–associated gene allele frequencies were the same or higher in populations of Latino or African descent.

**Meaning:**

Patients of racial/ethnic minorities are being underdiagnosed for leukodystrophies, which can result in a lack of treatment or insufficient treatment.

## Teaser

This case-control study compares the likelihood of being diagnosed with a leukodystrophy among US pediatric and adolescent patients of different racial/ethnic backgrounds and examines the prevalence of genes associated with leukodystrophy among racial/ethnic groups to investigate whether there are disparities in leukodystrophy diagnosis in different racial backgrounds.

## Introduction

Inherited leukodystrophies are a group of diseases affecting myelin that cause significant morbidities and death in 1 of 3 patients by age 8 years.^1^ Because certain leukodystrophies are clinically distinct, are uniquely diagnosable, and are the subject of extensive outcome and genetic studies, they have the potential to serve as an important proxy for understanding issues related to diagnosis and treatment in pediatric neurological and orphan diseases. Furthermore, children with neurological impairment, including leukodystrophies, account for a large and growing proportion of children’s hospital charges (30%).^2^

Over the past 2 decades, estimates of the incidence of leukodystrophies have been performed with varying methods at tertiary referral centers and population-based medical centers, as well as using national databases.^1,3,4,5,6,7,8,9,10,11,12^ However, a major limitation is that almost all studies of disease prevalence, outcomes, burden, and treatment have been performed in the United States or Europe, with patients drawn from predominantly Caucasian ancestry.^1,3^ To our knowledge, the incidence of leukodystrophies in racial/ethnic minority patient populations has not been examined, and it is not known whether diagnosis rates are equivalent in racial/ethnic minority populations, whether mutation rates are similar, and whether disease presentation or response to treatment differ. Because some leukodystrophies are treatable but require timely diagnosis and have therefore been added to newborn screening,^13^ understanding ethnic and racial differences is critical for effective screening, diagnosis, and treatment. Our objective was to determine whether there are disparities in leukodystrophy diagnosis in different racial backgrounds.

## Methods

This study follows the Strengthening the Reporting of Observational Studies in Epidemiology (STROBE) reporting guideline for cohort analyses.^14^ The study, which used deidentified data, was not considered human subjects research and was exempted by the institutional review boards at the University of Utah and Intermountain Healthcare. We conducted a retrospective case-control study on patients aged 18 years or younger admitted to a Pediatric Health Information System (PHIS) hospital with an *International Classification of Diseases, Tenth Revision (ICD-10)* diagnosis of leukodystrophy (metachromatic leukodystrophy [E75.25], X-linked adrenoleukodystrophy [E71.52x], Krabbe disease [E75.23], or Hurler disease [E76.01]) between October 1, 2015, and September 30, 2017.

The PHIS database has information from more than 50 children’s hospitals in the United States.^15^ Hospitals are affiliated with the business alliance of Child Health Corporation of America. Each patient has a unique entry, but the data are deidentified. Data collected include patient and visit demographic characteristics such as age, sex, race, insurance type, type of visit, estimated total visit cost, length of stay, *ICD* coding, and detailed charge information. The hospital visits included primarily inpatient admissions with additional visit types such as emergency department, observation, ambulatory surgery, and clinic visits. Race and ethnicity were collected as patient- or family-reported answers to separate questions at registration: 1 for race and 1 for ethnicity.

### Statistical Analysis

Patients with leukodystrophies were identified from any PHIS visit (inpatient, observation, emergency department, ambulatory surgery, clinic visit) as having an *ICD-10* code for 1 of the 4 leukodystrophies between October 1, 2015, and September 30, 2017. The control PHIS cohort of patients without leukodystrophy consisted of all other patients identified during the same period with an *ICD-10* code for diseases other than leukodystrophy.

We estimated the 2-year point prevalence of composite and individual leukodystrophy diagnoses in racial groups using a generalized linear model with a binomial distribution and logit linkage function. We fit the model with generalized estimating equations to account for correlation between patients within the same hospital. We estimated unadjusted and adjusted point estimates. Adjusted prevalence estimates were obtained by controlling for sex, insurance, urban or rural status, 2010 median household income for patient zip code, number of inpatient days, and age at first PHIS visit. All analyses were 2-sided, and *P* < .05 was considered statistically significant. Statistical analyses were conducted with SAS statistical software version 9.4 (SAS Institute Inc).

To compare leukodystrophy gene allele frequencies in different racial groups, we calculated missense and loss-of-function frequencies using the gnomAD database^16^ for *ABCD1*, *ARSA*, *GALC*, and *IDUA*. We used a Kruskal-Wallis test to determine significance with subsequent χ^2^ analysis.

## Results

We identified 557 patients with leukodystrophies (221 [40%] female; median [range] age, 7 [0-18] years) in the PHIS database over the 2-year period. The most common racial groups represented were white non-Hispanic (321 patients [58%]), black non-Hispanic (54 patients [10%]), and white Hispanic (51 patients [9%]) (Table 1). Patients had metachromatic leukodystrophy (MLD) (*ARSA* gene; 139 patients [25% of cohort]), X-linked adrenoleukodystrophy (*ABCD1*; 111 patients [20%]), Krabbe disease (*GALC*; 56 patients [10%]), or Hurler disease (*IDUA*; 56 patients [10%]). We chose these 4 *ICD-10* leukodystrophy codes because they provided specificity of an individual leukodystrophy genetic diagnosis. In contrast, other leukodystrophies coded using different *ICD-10* codes, such as sphingolipidoses, are nonspecific and include a large range of genetically discrete disorders, such as vanishing white matter disease, Canavan disease, and others.

**Table 1.** Demographic Characteristics of the Study Cohort

Characteristic	No. (%)	
	Patients With Leukodystrophy (n = 557)	Patients Without Leukodystrophy (n = 6 111 721)
Sex		
Male	336 (60)	3 231 564 (53)
Female	221 (40)	2 879 341 (47)
Race		
White non-Hispanic	321 (58)	2 632 374 (43)
Black non-Hispanic	54 (10)	1 191 477 (20)
White Hispanic	51 (9)	880 495 (14)
Asian	9 (2)	152 338 (2)
Black Hispanic	1 (0.2)	51 371 (0.8)
American Indian	3 (0.5)	14 417 (0.2)
Pacific Islander	1 (0.2)	10 533 (0.2)
Multiple	57 (10)	588 517 (10)
Other	46 (8)	413 292 (7)
Missing	14 (3)	176 907 (3)
Age, median (range), y	5 (0-18)	7 (0-18)

Nonwhite race/ethnicity, including black, black Hispanic, and white Hispanic background, was associated with not having a leukodystrophy diagnosis (Table 2). The adjusted prevalence for a leukodystrophy diagnosis in white non-Hispanic patients was 13.8 (95% CI, 10.6-17.9) per 100 000 patients, compared with 5.8 (95% CI, 3.8-8.9), 2.4 (95% CI, 1.1-5.2), and 7.4 (95% CI, 5.2-10.4) per 100 000 in black non-Hispanic, black Hispanic, and white Hispanic patients, respectively. This reduced rate of diagnosis was out of proportion to the frequency of the different races in PHIS. When each leukodystrophy was singly tested, nonwhite race was also associated with not having a diagnosis (Table 2).

**Table 2.** Adjusted Joint Prevalence of a Leukodystrophy Diagnosis in Different Racial Groups

Race	Combined Leukodystrophy Diagnoses		ALD		Hurler Disease		Krabbe Disease		MLD	
	Adjusted Prevalence, No./100 000 Patients (95% CI)^a^	*P* Value	Unadjusted Prevalence, No./100 000 Patients (95% CI)	*P* Value	Unadjusted Prevalence, No./100 000 Patients (95% CI)	*P* Value	Unadjusted Prevalence, No./100 000 Patients (95% CI)	*P* Value	Unadjusted Prevalence, No./100 000 Patients (95% CI)	*P* Value
White non-Hispanic	13.8 (10.6-17.9)	Reference	2.4 (1.9-3.1)	Reference	6.3 (5.4-7.3)	Reference	1.3 (1-1.9)	Reference	2.2 (1.7-2.8)	Reference
American Indian	23.9 (8.2-69.5)	.32	0 (0-100 000)	>.99	6.9 (1.0-49.2)	.92	0 (0-100 000)	>.99	13.9 (3.5-55.4)	.01
Asian	7.3 (3.2-16.3)	.10	0.7 (0.1-4.7)	.20	2.6 (1.0-7.1)	.09	0.7 (0.1-4.7)	.49	2 (0.6-6.1)	.85
Black Hispanic	2.4 (1.1-5.2)	<.001	1.9 (0.3-13.8)	.84	0 (0-100 000)	>.99	0 (0-100 000)	>.99	0 (0-100 000)	>.99
Black non-Hispanic	5.8 (3.8-8.9)	<.001	1.3 (0.8-2.2)	.04	1.2 (0.7-2.0)	<.001	0.4 (0.2-1.0)	.02	1.6 (1.0-2.5)	.22
Multiple	12.3 (8.3-18.3)	.55	3.9 (2.6-5.9)	.04	3.7 (2.5-5.7)	.02	0.7 (0.3-1.8)	.20	1.4 (0.7-2.7)	.20
Other	10.6 (6.0-18.9)	.32	1.5 (0.7-3.2)	.24	2.9 (1.6-5.1)	.01	2.7 (1.5-4.8)	.05	4.1 (2.6-6.6)	.02
Pacific Islander	12.3 (1.9-79.9)	.90	0 (0-100 000)	>.99	9.5 (1.3-67.4)	.68	0 (0-100 000)	>.99	0 (0-100 000)	>.99
White Hispanic	7.4 (5.2-10.4)	<.001	1.9 (1.2-3.1)	.43	2.3 (1.5-3.5)	<.001	0.7 (0.3-1.5)	.13	0.9 (0.5-1.8)	.02

Abbreviations: ALD, X-linked adrenoleukodystrophy; MLD, metachromatic leukodystrophy.

^a^ Adjusted point prevalence for the combined leukodystrophy diagnoses controls for sex, insurance type, urban or rural status, 2010 median household income from 2010 US Census data for most common patient zip code at Pediatric Health Information Systems visits, number of inpatient days for the patient for all admissions during the study, and patient age.

To explore whether lower leukodystrophy gene allele frequencies in nonwhite races might account for lower diagnosis rates, we determined the frequency of missense and loss-of-function alleles in *ABCD1*, *ARSA*, *GALC*, and *IDUA* (Figure) using the gnomAD database.^16^ We observed similar or higher frequencies of missense or loss-of-function alleles in populations of Latino and African descent. For *ABCD1*, allele frequencies in those of Latino or African descent were 2.1 × 10^−5^ and 2.2 × 10^−5^, as compared with 1.4 × 10^−5^ for those of European non-Finnish descent. For *ARSA, GALC,* and *IDUA,* allele frequencies in those of Latino or African descent were 2.7 × 10^−3^ and 3.9 × 10^−3^ vs 3.1 × 10^−3^, 4.1 × 10^−3^ and 5.6 × 10^−3^ vs 5.0 × 10^−3^, and 4.8 × 10^−3^ and 8.0 × 10^−3^ vs 5.3 × 10^−3^.

**Figure.**
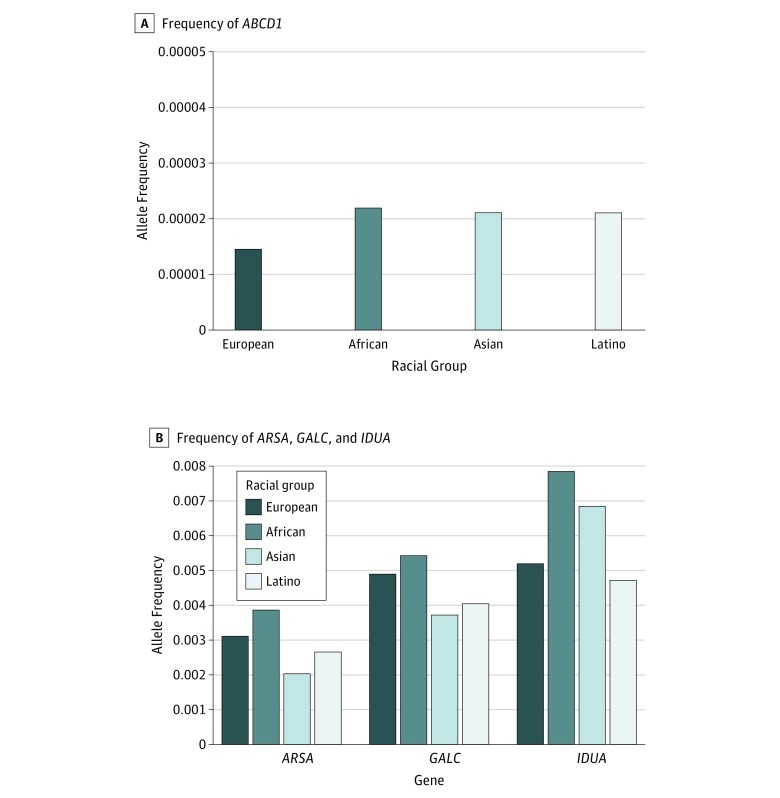
Missense and Loss-of-Function Allele Frequencies in Different Racial Populations A, *ABCD1* allele frequencies. B, *ARSA*, *GALC*, and *IDUA* allele frequencies. For all gene alleles, comparison of each racial population to European non-Finnish population was significant (*P* < .05).

## Discussion

We found that patients of racial/ethnic minorities, including those from black, black Hispanic, and white Hispanic backgrounds, were significantly less likely to be diagnosed with a leukodystrophy. Furthermore, we observed that leukodystrophy disease–associated allele frequencies were the same or higher in populations of Latino or African descent.

There are known examples of genetic founder effects for specific leukodystrophies in some populations. For example, there is a single historical origin of the mutation in *ARSA* causing MLD in patients of Arabic heritage^17^ and there is a single origin from the 19th century causing a higher rate of MLD in the western Navajo Nation.^18^ However, our observation of comparable or higher frequencies of disease gene alleles in different racial backgrounds suggests that a genetic founder effect is unlikely to account for the observed differences in diagnosis.

Strengths of this study are the use of specific *ICD-10* codes to track specific leukodystrophy diseases. In the past, tracking outcomes for patients with leukodystrophies has been more difficult because *ICD-9* codes have a relatively low specificity to the diagnosis of an inherited leukodystrophy, and relatively complex algorithms had to be used.^19^ With the use of *ICD-10* codes for X-linked adrenoleukodystrophy, MLD, Krabbe disease, and Hurler disease, we were able to track and compare specific leukodystrophies, which would not have been possible using *ICD-9* coding or other less specific *ICD-10* codes. Finally, the use of the PHIS national database provides a more comprehensive view of trends across the United States for these relatively rare diseases.

### Limitations

Limitations for this study are its use of retrospective data and the limited duration of data collection. The time limitation was necessary because *ICD-10* has only been in use for the past 3 years, and *ICD-10* code use was necessary to have sufficient specificity in determining diagnoses. We cannot exclude the possibility that the differences observed in diagnosis were related to different rates of hospital admission. For example, perhaps black patients with leukodystrophies were admitted less often to the hospital, or their families did not bring them to the emergency department as often. Another limitation is that PHIS consists primarily of in-hospital data, with less outpatient clinic data, so it is not possible to accurately determine population rates of disease or prevalence. Our analysis of leukodystrophy gene allele frequencies used the data deposited in gnomAD, which may not have the same racial group distributions as PHIS. Further work should be done to better understand the nonwhite distribution of patients with leukodystrophies; because many patients with leukodystrophies report *multiple* for racial categorization, it is unclear whether this reflects interracial heritage or whether it is due to issues inherent in data collection.

## Conclusions

The reason for the disparity in leukodystrophy diagnosis is not clear. Causes could include differences in de novo mutation rates,^12^ the presence of disease modifier genes, different clinical symptoms in different racial backgrounds, incorrect diagnosis, or lack of access to specialists or to coverage for testing. This underdiagnosis has implications for newborn screening programs and treatment access because false-positive and false-negative rates affect sensitivity and specificity of screening. Furthermore, as our work is, to our knowledge, the first of its kind in pediatric neurological diseases, we are concerned that the findings of underdiagnosis may reflect a more widespread problem in pediatric neurological and orphan diseases.
